# Retraction Note: Dysregulation of ghrelin in diabetes impairs the vascular reparative response to hindlimb ischemia in a mouse model; clinical relevance to peripheral artery disease

**DOI:** 10.1038/s41598-026-59522-7

**Published:** 2026-06-30

**Authors:** Joshua P. H. Neale, James T. Pearson, Kate N. Thomas, Hirotsugu Tsuchimochi, Hiroshi Hosoda, Masayasu Kojima, Takahiro Sato, Gregory T. Jones, Adam P. Denny, Lorna J. Daniels, Dhananjie Chandrasekera, Ping Liu, Andre M. van Rij, Rajesh Katare, Daryl O. Schwenke

**Affiliations:** 1 Department of Physiology, School of Biomedical Sciences and HeartOtago, University of Otago, 270 Great King Street, Dunedin, 9018 New Zealand; 2 Department of Cardiac Physiology, National Cerebral and Cardiovascular Center Research Institute, Suita, Osaka Japan; 3 Biomedicine Discovery Institute and Department of Physiology, Monash University, Clayton, Australia; 4 Department of Surgical Sciences, University of Otago, Dunedin, New Zealand; 5 Department of Regenerative Medicine, National Cerebral and Cardiovascular Center Research Institute, Suita, Osaka Japan; 6 Molecular Genetics, Institute of Life Science, Kurume University, Kurume, Japan; 7 Department of Anatomy, School of Biomedical Sciences, University of Otago, Dunedin, New Zealand

Retraction of: *Scientific Reports* 10.1038/s41598-020-70391-6, published online 12 August 2020

The Editors have retracted this Article.

After publication, concerns were raised regarding some of the data presented in the figures, specifically:

- Fig. 4A ND D0 AG and DAG images appear highly similar;
- Fig. 4E DM Veh and DAG images appear highly similar;
- Fig. 5A DM Sham and DAG images appear highly similar;
- Fig. 5E AG and DAG images appear to overlap;
- Fig. 5E DM Sham and AG images appear to overlap.

The Authors provided the underlying raw data for validation; however, further checks by the Editors found discrepancies between the raw data and published figures.

The Editors therefore no longer have confidence in the results of this Article.

James T. Pearson, Hirotsugu Tsuchimochi, Takahiro Sato and Gregory T. Jones agree with this retraction. Daryl O. Schwenke has not explicitly stated whether they agree with this retraction. The other authors have not responded to any correspondence from the Editors about this retraction. The Editors have not been able to obtain current email addresses for Hiroshi Hosoda and Lorna J. Daniels.

